# Web Service Reputation Evaluation Based on QoS Measurement

**DOI:** 10.1155/2014/373902

**Published:** 2014-04-13

**Authors:** Haiteng Zhang, Zhiqing Shao, Hong Zheng, Jie Zhai

**Affiliations:** Department of Computer Science and Engineering, East China University of Science and Technology, Shanghai 200237, China

## Abstract

In the early service transactions, quality of service (QoS) information was published by service provider which was not always true and credible. For better verification the trust of the QoS information was provided by the Web service. In this paper, the factual QoS running data are collected by our WS-QoS measurement tool; based on these objectivity data, an algorithm compares the difference of the offered and measured quality data of the service and gives the similarity, and then a reputation evaluation method computes the reputation level of the Web service based on the similarity. The initial implementation and experiment with three Web services' example show that this approach is feasible and these values can act as the references for subsequent consumers to select the service.

## 1. Introduction

Nowadays, Web services are one of the important innovations in software which bring many benefits in software design and implementation. With the fast growth of Web services, a large number of Web services with the same or similar function are developed and released. How to select a suitable and best service has become an important research topic. The Web service selection technology based on QoS has been referred to for solving this problem, which considers distinguishing those Web services with the same function using a set of different QoS levels [1].

The existing QoS-based services selection approaches always assume that the QoS data coming from service providers are effective and trustworthy. However, the values of QoS attributes which are provided by service providers may be incredible, since service providers sometimes may advertise higher QoS data than the factual level of the service in order to attract more users to use their services and so gain better benefits [2]. For example, the maximum response time of these services may be increased, while the invocation rate remains under a certain threshold during runtime. Therefore, how to give the objective and effective evaluation to service provider's reputation to help the consumer to reference and choose the appropriate service becomes a problem to solve [3].

To ensure the impartiality and objectivity of a Web service reputation evaluation, this paper proposes a trust Web service reputation evaluation framework based on QoS similarity of the factual values and the advertised values. Firstly, a Web services QoS measurement tool which is independent of service providers or consumers was developed, which provides an automatic approach on measuring and storing QoS values of the service. Secondly, Web service reputation evaluating component computes the similarity of advertised QoS values and factual values, and then the similarity is used to evaluate the reputation level of the Web service. Lastly, a set of experiments are given, which show that this approach can effectively evaluate the reputation of the service provider and thus can strengthen the effectiveness of the service selection.

The rest of this paper is organized as follows. In Section 2, we give an overview of our Web service reputation evaluation framework.  Section 3 illustrates the core component of our WS-QoS measurement tool. The similarity algorithm and QoS reputation evaluation method are given in Section 4. In Section 5 we present the main implementation and the experiment to verify the efficiency of our method. This is followed by an overview of the related work in Section 6.  Section 7 concludes our paper and presents further research directions.

## 2. The Web Service Reputation Evaluation Framework

**Figure 1:**
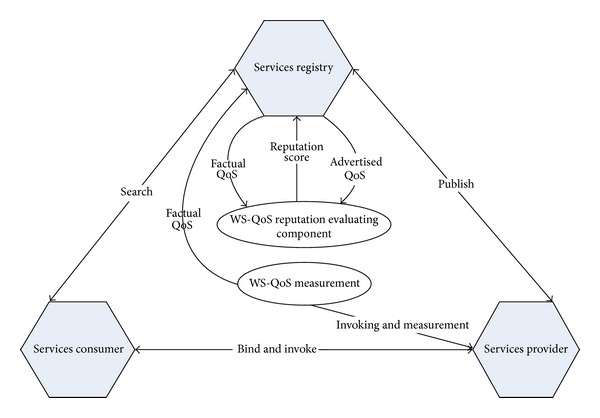
The Web service reputation evaluation framework.

The Web service reputation evaluation framework is shown in Figure 1. The framework consists of the basic Web service model components like the Web service provider, Web service consumer, and the Web service registry. Two major components are introduced into traditional Web services architecture to realize the reputation evaluation of the service provider.

WS-QoS measurement tool is a client side technique which works completely on Web service consumer and provider independently. It measures the performance related QoS values which are achieved by dynamic invoking Web services together with aspect-oriented programming (AOP). So that factual QoS values are provided and stored by this component.

WS-QoS reputation evaluating component supports service reputation measurement based on QoS similarity. Service providers issue the advertised values of the QoS information into Service Registry Center. WS-QoS measurement also gives feedback of the factual values of QoS to Service Registry Center after invoking the service. QoS similarity is computed firstly according to the differences between advertised QoS and factual QoS values, and then the Web service reputation score was given based on these similarities.

## 3. The WS-QoS Measurement Tool

**Figure 2:**
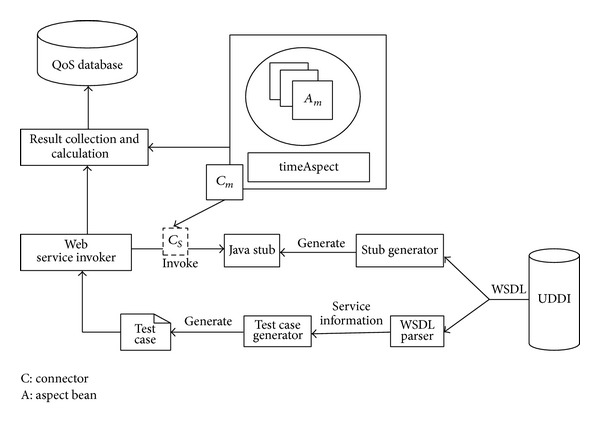
The architecture of the WS-QoS measurement tool.

To objectively measure service related quality information, the WS-QoS measurement tool is designed to acquire QoS attribute values for a given set of Web services. The main processes of the WS-QoS measurement tool are depicted in Figure 2. In the first phase, Web services description language (WSDL) file is acquired from UDDI. The WSDL file is parsed to get service related information, and the test data are generated for each input element of the operations. As a next step, the Web service stub classes are generated as Java files by using the WSDL2Java tool from Axis, which gives the service invoker all the exposed methods and parameters' types by the Web service. In the third step, the Web service invoker assembles the generated test date to stub code to cause the Web service to be invoked and its response results and status to be collected which can be used to compute the QoS typical parameters such as availability, reliability, and accessibility. In the last step, timeAspect code weaves time measurement codes before and after the byte code of the Web service invoking method; then the start time and end time of Web service call are acquired, and the Web service response time is computed.

### 3.1. Test Case Generation Based on WSDL

In the distributed environment, the service provider exposes the functionality of the service in the form of a Web services description language. WSDL describes Web services by using the following major elements: portType, message, parts, types, binding, port, and service [4]. For Web services dynamical invocation, WSDL parser is first needed to get service related information such as service name, description, operations, and the data type of the input arguments and the output arguments. WSDL4J has been used to parse the WSDL files by many Web services underlying technology implementations [5]. To obtain a complete Web service information needed to invoke the service, this technology is also used in our component. The parser reads the WSDL specification and extracts the operation and the message tags that are exposed by a particular Web service from the WSDL; in this way the methods and their input arguments' and return arguments' types of the service are acquired which will later on help the Web service invoker in invoking the required method of that service.

Then a test case knowledge base is established based on these pieces of information, as described in [6, 7], where each simple data type is associated with default facets definition and sets of candidate values based on the test strategies such as random values and boundary values. Complex data type defines a composition of simple and/or complex data types. To generate test data of complex data types, the generator recursively analyzes the structure of the data type until it reaches the simple type. The generated service information and test cases are documented in XML-based test files, which can be easily used by service invoker.

### 3.2. Web Service Stubs Generation Based on WSDL

Stubs are client side programs which act as a proxy for the server. Stubs are used to make calls to the Web services. Using stubs simplifies our applications considerably. We do not have to write a complex client program that dynamically generates SOAP requests and interprets SOAP responses. We can simply concentrate on writing the Web service invoking client code and leave all the other work to the stubs. The stubs are generally generated only once and then we can reuse the stubs as many times as we want. WSDL2Java from Axis is a tool that generates Java classes from an existing WSDL document. Generated classes represent a service and port combination with operations as methods. A data type class represents an input or output message part [8].

WSDL2Java generates the following stub and skeleton classes from existing WSDL documents: (1) the data type class that represents the input message part defined in the WSDL document; (2) the data type class that represents the output message part defined in the WSDL document; (3) the stub class that represents a combination of service and port defined in the WSDL document; (4) the default constructor method to create a stub object with information defined in the WSDL document; (5) the stub method that represents an operation defined in the WSDL document.

### 3.3. The Web Service Invoker

WSDL2Java analyzes WSDL file of Web service and creates the stub program and some interface programs. However we have to create the client program to execute the Web service by composing those stub and interface programs. Therefore, the Web service invoker is developed which tries to invoke a service operation just by “probing” arbitrary test values for the input parameters for an operation. Firstly the Web service invoker analyzes the Java code using “Class” and “Method” API in Java reflection and we can get the getter method and its return type. Secondly, the information from the test case file is acquired and some parameters required for the dynamic invocation of Web service are set in our system. Thirdly, Java reflection is used to dynamically instantiate these complex classes and Web service stubs. By dealing with the transactions described above, the Web service's operation is executed. Lastly, responses' results and status of the Web service are analyzed and collected by Result Collector and Calculation Component and stored in the QoS database. ome computation models of QoS properties are given in [9], which can be computed on the basis of the Web service invoker results and status.

### 3.4. The timeAspect

Response time is the time needed to process a query, from the moment of sending a request until receiving the response [9]. For measuring Web service response time, before sending the request, the current date and time are saved, and after receiving the response from Web service, the date and time are saved again. The response time of the Web method is calculated by subtracting the sending request time from the receiving response time. For keeping flexibility, this paper proposes using aspect-oriented programming technology to measure the response time of Web services. AOP approaches introduce a new concept to modularize crosscutting concerns, called an aspect. An aspect defines a set of join points in the target application where the normal execution is altered. Aspect weavers are used to weave the aspect logic into the target application [10]. The goal of AOP is to achieve a better separation of concerns, which makes it particularly suitable for solving the problems of Web services response time collection. This is because time record part is such a crosscutting concern since it spans over each service we have to invoke. Before the service invoking method starts execution, timeAspect points to the codes and records the start time; after the service invoking method is performed, timeAspect also weaves the codes and records the end time. The response time is equal to subtracting the start time from end time.

## 4. WS-QoS Reputation Evaluation

In order to provide better evaluation of the service provider's reputation, this section gives the computing model of the Web service's QoS similarity. The values of similarity can be used to represent the reputation level; higher values of similarity represent a better reputation level.

### 4.1. The Calculation of the Global QoS

For each call to the service by WS-QoS measurement tool, the collected Web services QoS attribute values may be different. In order to be able to reflect the real property of the dynamic changes of QoS, global QoS value of the service must be recomputed and stored based on the historical data and current data.

Given a Web service, it has m QoS attributes can be expressed as *A* : {*A*_*j*_, 1 ≤ *j* ≤ *m*}. The Web service QoS attribute values set is defined as *Q* : {*q*_*j*_, 1 ≤ *j* ≤ *m*}; *q*_*j*_ is the attribute value of the attribute *A*_*j*_. The *n*th current factual QoS data collected by the WS-QoS measurement tool is defined by the set fa_*Q*_*n*_ : {fa_*q*_*nj*_, 1 ≤ *j* ≤ *m*}; fa_*q*_*nj*_ is the *n*th actual value of the attribute *A*_*j*_. Then the global QoS value of the service can be computed by using two-time average method. We randomly sample *p*(*p* ≤ *n*) numeric from the *n* values (*p* ≤ *n*); the *l*th QoS value fa*_q*_*lj*_ can be computed as in formula (1), so sample *k* times can get *k* sampling value, and then the global QoS value of the service fa_*Q*_*g*_ = {fa_*q*_*j*_, 1 ≤ *j* ≤ *m*},fa*_q*_*j*_ can be computed as in formula (2):

(1) $$\begin{array}{c} \text{fa}\_q_{lj}=\frac{1}{p}\overset{p}{\underset{i=1}{\sum }}\text{fa}\_q_{ij},\;p\neq 0, \end{array}$$

(2) $$\begin{array}{c} \text{fa}\_q_{j}=\frac{1}{k}\overset{k}{\underset{l=1}{\sum }}\text{fa}\_q_{lj},\;k\neq 0. \end{array}$$

### 4.2. The Calculation of the QoS Similarity

Similarity is acquired by calculating the accumulation average of the comparative result between the advertising quality values and the factual global quality values. QoS attributes hold two different directions or tendencies of their values; if the tendency of the attribute is positive, it means that a bigger value is better. On the contrary if the tendency is referred to as negative, it means that smaller values are preferred. For example, for attribute “response time” the smaller value is usually preferred, so the tendency of this parameter is negative, whereas for attribute “availability” the bigger value indicates a better quality for the specified parameter, so the tendency is positive. Based on the direction of the attribute, the similarity can be computed by the following formulas (3)–(5). As described in Section 4.1, the global QoS value of the service is fa_*Q*_*g*_ *and *fa_*Q*_*g*_ = {fa_*q*_*j*_, 1 ≤ *j* ≤ *m*}; the advertised QoS which reflects the quality offered by the Web service provider is defined by the set ad_*Q* = {(ad_min_*q*_*j*_, ad_max_*q*_*j*_), 1 ≤ *j* ≤ *m*}. ad_min*_q*_*j*_ and ad_max*_q*_*j*_ refer to the minimum value and the maximum value of the advertised quality attribute *A*_*j*_, respectively. Consider

(3) $$\begin{array}{c} \text{sim}=\frac{\sum_{j=1}^{m}c}{m}. \end{array}$$

If the tendency of attribute is negative,

(4) $$\begin{array}{c} c=\{\begin{array}{cc} 0 & \text{if}\;{\text{fa}\_q}_{j}\leq \text{a}{\text{d}\_\text{max⁡}\_q}_{j}\\ 1 & \text{if}\;{\text{fa}\_q}_{j}>{\text{ad}\_\max\_q}_{j}. \end{array} \end{array}$$

If the tendency of attribute is positive,

(5) $$\begin{array}{c} c=\{\begin{array}{cc} 0 & {\text{if}\;\text{fa}\_q}_{j}\leq \text{a}{\text{d}\_\min\_q}_{j}\\ 1 & \text{if}\;{\text{fa}\_q}_{j}>\text{a}{\text{d}\_\min\_q}_{j}. \end{array} \end{array}$$

### 4.3. The Evaluation of the QoS Reputation Level

The QoS similarity obtained by using the aforementioned methods is between 0 and 1 (0 ≤ sim ≤ 1). The interval is divided into 5 stages, that is, [0, 0.2], [0.2, 0.4], [0.4, 0.6], [0.6, 0.8], and [0.8, 1]; each stage can correspond to a reputation level. According to the rank, the reputation level is ordered from low to high, respectively, that is, 1,2, 3,4, 5, which represent reputation scores of the service. It is shown that if sim is higher, then the difference between the factual value and the advertised value of QoS is smaller and the reputation score is higher and vice versa.

## 5. Implementation and Experiment

We choose Java-based open source platforms and tools to implement the measurement tool. Axis provides better support to call Java and Java-based service, so we use the Axis to develop the client invoker part and deploy the simulation Web service in Axis. For parsing and analyzing the WSDL files we use the WSDL4J library from SourceForge [5]. The transformation from WSDL to Java classes is handled by the Axis WSDL2Java tool. timeAspect code is implemented with AspectJ.

To demonstrate the validity of our approach, the following three Web services are used as a sample in our experiment: (1) getWeatherInformation: which allows you to get your city forecast over the next 7 days and is updated hourly; WSDL address is http://wsf.cdyne.com/WeatherWS/Weather.asmx?WSDL; (2) SendService: methods to send SMS messages individually and in batches; WSDL address is http://www.esendex.com/secure/messenger/soap/SendService.asmx?wsdl; (3) GlobalWeather: which gets country weather information; WSDL address is http://www.webservicex.com/globalweather.asmx?WSDL.

**Figure 3:**
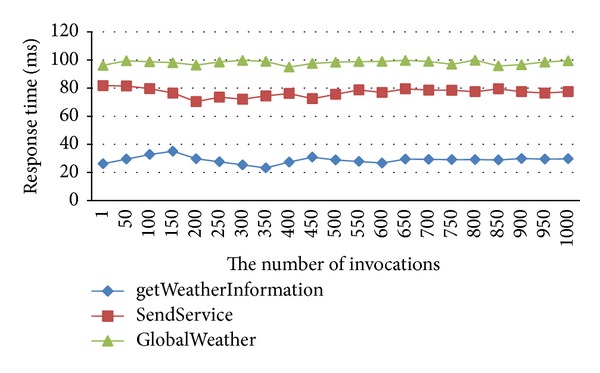
Global response time.

**Figure 4:**
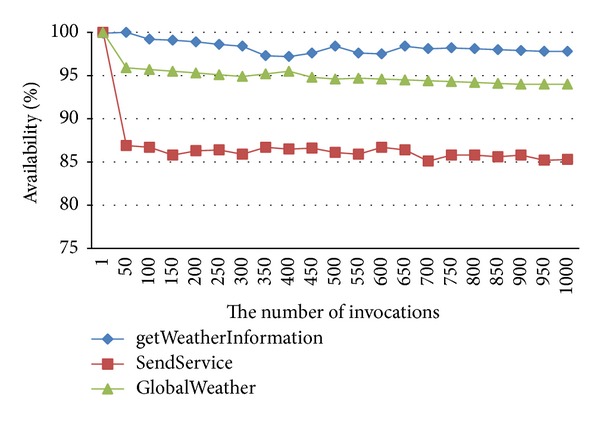
Global availability.

We invoked and monitored the mentioned services for 1000 times by using our tool, and then the factual QoS values such as response time, availability, and accessibility can be acquired. Figures 3-4 give changes of the global response time and availability with the difference of the invoking times. From the figure we can see that the global QoS values are more stable by using two-time average method with the increasing of the invoking times. In addition, Even if only 50 calls, we can still achieve good results. The experiment proved that our QoS measurement tool is useful and the calculation method of the global QoS is feasible; the global QoS value can fully represent the factual QoS value.

Table 1 gives the similarity between the factual values and the advertised values and shows the reputation level of the three Web services. From this table, we can see that not only our approach gives the reputation level objectively, but also similarity can be used as a rank of Web service, which can be useful to help service consumer select service.

## 6. Related Works

Artaiam and Senivongse [11] review a QoS model which covers various dimensions of service quality (i.e., availability, accessibility, performance, reliability, security, and regulatory) and propose metrics to enhance QoS measurement on the service side. A monitoring tool is designed and developed as an extension to Web services monitoring feature of Java system application server under Sun's Glass Fish project. Chen et al. [12] propose a novel trustable mechanism to monitor and evaluate SLA compliance based on the AOP paradigm. Authoritative monitoring aspects are supplied by a trustable SLA manager and by weaving the aspects into susceptible service runtime; service providers are ensured to monitor and report their service status obligatorily and accurately. In contrast, our WS-QoS measurement tool is a client tool and measures QoS values standing in the position of customers.

Michlmayr et al. [9] present a framework that combines the advantages of client and server side QoS monitoring. It builds on event processing to inform interested subscribers of current QoS values and possible violations of service level agreements. Rosenberg et al. [13] present an evaluation approach for QoS attributes of Web services, which works completely on service and provider independently; it also assesses performance of specific values (such as latency or service processing time) that usually require access to the server which hosts the service. Their approach is similar to ours, but they omit a way to specify how QoS test parameters' values are generated.

Nonintrusive monitoring [14–16] requires the establishment of mechanisms for capturing runtime information on service execution, for example, service operation calls and responses. In this way, monitoring logic is responsible for evaluation service QoS. This paper also employs aspect-oriented programming to ensure monitoring aspect codes separated from the service code. References [17–19] focus on the provision of a QoS monitoring architecture and measure QoS compliance in SOA infrastructures. Compared with our work, it is not specified how QoS attributes are actually measured.

Kalepu et al. [3] consider that the reputation of Web service consists of user ranking, compliance, and verity. They measure the consistency in service providers to deliver the QoS level specified in their contracts, which has been proposed as a metric to evaluate the reputation of Web services. According to the paper's proposal, we do an in-depth study and provide the concrete implementation. Fu et al. [2] design corresponding upper and lower QoS ontology for computing QoS consistency of factual value with advertised value automatically. The QoS consistency computing algorithm supports hierarchical QoS item consistency computing. Compared with our work, it is not specified how QoS values are actually measured. Nianhua et al. [20] propose a reputation evaluation algorithm for the new added Web service based on the similarity theory. Similarities and trust are used as weights for computing reputations from different recommenders. Zhao et al. [21] propose a gradually adjusting reputation evaluation method of Web services based on eliminating the collusive behaviors of consumers step by step, and a reputation-aware model for service selection is designed. Unlike us, the reputation score is computed based on subjective judgment of service users but not objective measurement. Shao et al. [22] propose a similarity computing algorithm for Web services and their consumers based on Euclidean distance theory. Consumers' similarities are used as weights of indirect experiences. However, their similarity computing algorithm is different from us and mainly used in the QoS comparison between service providers and service consumers. Jøsang et al. [23] combine Bayesian reputation systems with a trust model for evaluating the quality of service in a single framework. Nepal et al. [24] propose a fuzzy trust evaluation approach for Web services. Both of them pay attention to propose a trust and reputation management framework for Web service selection.

## 7. Conclusions

This paper gives the factual QoS values by using our QoS measurement tool, compares the similarity of the factual QoS values and advertising QoS values, and completes the impartiality and objective Web service reputation evaluation. WS-QoS measurement tool is implemented by dynamically invoking the Web services and weaving aspects code into the Web service invoking code. Similarity is acquired by comparing the advertising quality values and the global quality values. According to the similarity, the reputation level is ordered from low to high. By a set of experiments, we prove the effectiveness and feasibility of the method. In the future, we will consider improving the QoS measurement tool, supporting more runtime data acquisition; furthermore, we plan to research on the updating algorithms for trust and reputations, making trustworthiness information reflect the latest changes in service.

## Figures and Tables

**Table 1:** Similarity and reputation level.

QoS attribute/service name	getWeatherInformation	SendService	GlobalWeather
Adv_Response (ms)	(30, 40)	(40, 60)	(80, 95)
Fac_Response (ms)	38.9	76.9	98.1 ms
Adv_Availability	(98%, 100%)	(80%, 90%)	(90%, 100%)
Fac_Availability	97.2%	87.5%	94.8%
Adv_accessibility	(80%, 100%)	(85%, 90%)	(90%, 100%)
Fac_accessibility	88.6%	82.4%	93%
Similarity	1	0.33	0.67
Reputation level	5	2	4
